# Strength and Failure Analysis of Fiber-Wound Composite Gas Cylinder via Numerical Simulation

**DOI:** 10.3390/ma17030717

**Published:** 2024-02-02

**Authors:** Xiaodi Wu, Bo Yang, Song Zhou

**Affiliations:** 1 School of Marine Engineering and Technology, Sun Yat-Sen University, Zhuhai 519082, China; wuxd23@mail.sysu.edu.cn; 2 Shanghai Institute of Special Equipment Inspection and Technical Research, No. 915 Jinshajiang Road, Putuo District, Shanghai 200062, China; 3 College of Civil Aviation, Northwest Polytechnical University, Taicang 215400, China

**Keywords:** Hashin criterion, failure analysis, carbon fiber, filament-wound composite gas cylinder

## Abstract

Based on the classical grid theory and related regulations, a structure model of a fiber-wound composite gas cylinder was designed in this paper. Based on the design results, a finite element model of a fully wound composite cylinder of an aluminum alloy inner liner with a working pressure of 35 MPa was established based on the ABAQUS software, and its stress distribution under working pressure and minimum burst pressure was analyzed. According to engineering experience, the pressure tolerance of composite cylinders can be improved by proper autofrettage pressure before working pressure, so the influence of autofrettage pressure was analyzed in this paper. The optimum autofrettage pressure was selected by setting the autofrettage gradient, and damage analysis was carried out on the cylinder with nominal working pressure of 35 MPa based on the Hashin failure criterion. The results show the initial damage sequence: matrix stretching occurs before the fiber stretching, and the damage generally starts from the spiral-wound layer. The tensile damage first appears in the transition section between the head and the barrel body, and the damage of the spiral-wound layer develops from the inner layer of the wound layer to the outer layer, while the damage of the circumferentially wound layer develops from the outer layer to the inner layer.

## 1. Introduction

A gas cylinder [1,2,3,4,5] is a mobile container for storing compressed gas or liquefied gas. In the past, it was usually made of high-strength metal materials such as steel and aluminum alloy by pressing and drawing, pipe closing and other methods. There are different sizes according to the required volume size and set pressure, but metal materials are usually dense, and when the required volume is large, the cylinder’s self-weight can reach thousands of tons, which seriously affects and restricts its development and application. Therefore, in order to ensure that the gas cylinder can withstand high pressure and reduce its own weight, the fiber-wound composite gas cylinder came into being. Fiber-wound composite gas cylinders combine the advantages of traditional pressure vessels and composite materials, which greatly improves the service range, efficiency and life of pressure vessels.

Fiber-wound composite gas cylinders [6,7,8,9,10,11,12] are usually composed of a thin metal liner with a certain thickness of resin-based fiber-reinforced composite. Among them, the metal lining materials are usually selected aluminum alloy, stainless steel and other materials, which only bear a small part of the pressure load, and the main function is to prevent gas leakage of the container in transportation. The composite material layer, as the main bearing part, bears most of the pressure load of the container (more than 75%) and usually uses materials such as carbon fiber, aramid fiber and glass fiber. In terms of fatigue performance, the cycle life of the fiber layer is much longer than that of the metal liner, so the fiber-wound composite cylinder has the failure mode of leakage before blasting that the metal cylinder does not have. At present, there are various designs of fiber-wound composite gas cylinders [13,14,15,16,17], which are widely used in rocket missiles [18,19], energy storage and transportation [20,21,22], new energy vehicles [4,23,24,25,26], industrial production [27,28,29], medical rescue [30,31] and other fields.

According to the classification of the winding process, there are three basic winding methods of fiber-wound composite gas cylinders [10]: circular winding, spiral winding and plane winding. According to ref [32], when the fiber layer is only circumferentially wound, its limited life will be shortened due to the high rupture pressure of the circumferential fiber layer. Therefore, better results can be obtained by changing the winding method and combining the circular winding and spiral winding. Therefore, for the structural design of composite gas cylinders, Chen [8] obtained the calculation method of the wall thickness and bursting pressure of the cylindrical cylinder of the fiber-wound shell in a solid rocket engine based on the grid theory and gave the method for determining the strength of the fiber by using the simulated experimental pressure vessel. The design of the dome of the cylinder is always a difficult point in the design of the cylinder model. The shape of the fiber layer at the dome has been studied by domestic and international researchers. Mei et al. [33] initially studied the dome thickness of fiber-wound composite pressure vessels. Liang et al. [34] optimized the dome shape of the fiber-wound composite pressure vessel based on the shape factor. Kim et al. [35] optimized the fiber winding structure based on the semi-geodesic path algorithm. Kumar S S et al. [36] found that the geodesic winding at the dome can prevent sliding and twisting between the fiber and the winding surface, which can ensure the stability of the structure. Through a series of explosion tests and structural analyses, Cho S M et al. [37] proved that the type 4 composite cylinder designed with equal ellipsoidal roof theory is safer in terms of failure mode. Zu L et al. [38] proposed a novel design approach to determine the optimal winding parameters of composite pressure vessels based on non-geodesic trajectories. Jois K C et al. [39] studied the stress distribution along cylindrical composite pressure vessels with different dome geometries. Sharma P et al. [40] analyzed the effects of dome shape on the burst pressure, failure characteristics and weight performance of the vessel. Padovec Z et al. [41] calculated analytical and numerical solutions for five selected domes (a spherical shell, a geodesicisotensoid shell, a shell with zero transversal strain, a shell with zero transversal stress and a shell with identical strain). The stresses and strains in the domes were evaluated analytically from known equations with the use of MATLAB script for numerical evaluation and via finite element analysis (FEA) with Abaqus software, and the results were compared with each other. In this paper, a method similar to Xiao’s [14] is adopted to develop the dome and assign different material properties to the unit.

As for the study of mechanical properties of composite cylinders, strength analysis is the research basis to ensure the normal operation of cylinders under working pressure, and many scholars have conducted a lot of research in this respect [42,43,44,45,46,47,48,49,50,51]. Among them, Hocine et al. [31] conducted an experimental and analytical study of the cylindrical part of a fiber-wound reinforced metal container under internal pressure. Sharifi S et al. [45] studied the effect of staking sequence and geometrical shape on the mechanical strength of the shell of a laminar woven composite. Pavan E et al. [47] found the effect of shell thickness on the burst pressure of composite pressure vessels. Magnucki K et al. [48] analyzed the strength of the dished heads of atypical pressure vessels under the condition of eliminating the edge effect arising at the joint of the cylindrical shell and the dished head. Kumar E et al. [49] used Ansys to model and analyze different stiffener designs in pressure vessels and recommended the best stiffener design by considering factors such as specific structural stiffness, von mises stress, weight and total deformation. Wang et al. [50] studied the stress distribution law of an aluminum alloy liner and wound composite layer under the conditions of autofrettage, zero pressure, working pressure, hydrostatic test pressure and minimum burst pressure. Lin J et al. [51] used the finite element method to analyze the strength of composite gas cylinders, and the research results were in good agreement with the results of a hydraulic blasting test. In the process of strength research and analysis, some scholars have found that autofrettage treatment of the cylinder before work can help the cylinder withstand higher working pressure. Son et al. [52] adopted layer-based modeling technology on the finite element model of composite gas cylinders and focused on the influence of autofrettage pressure. The stress analysis of the pressure vessel was carried out to determine the most suitable autofrettage pressure. Based on finite element analysis, Hu [53] and Zhang [54] determined the autofrettage pressure of carbon fiber-wound hydrogen storage vessel and further estimated the material failure on this basis. Enqi W et al. [55] found through experiments that under the optimum autofrettage pressure, the bearing capacity of the liner under working pressure and the fatigue life under fatigue cycle load were increased by 74% and 12 times, respectively.

In addition to meeting the strength conditions under working pressure, the cylinder design should also ensure that it can be used for a long time under the cyclic loading conditions of working pressure, so damage analysis of the cylinder is also widely paid attention to. The damage analysis of gas cylinders mainly considers the failure of composite materials. There are many kinds of failure analysis methods for composite materials according to different research purposes, including the finite element analysis method [56,57,58], phase field method [59,60,61], nondestructive detection method [62,63], etc. The finite element analysis method is used to simulate the real physical system (geometry and load conditions) by numerical approximation and discretization with the help of high-performance computer tools, such as solving the continuity problems of structure, heat conduction, electromagnetic field, damage mechanics, etc., which can be used to analyze the damage problems of composite materials. Onder A et al. [56] studied the rupture pressure of a wound composite pressure vessel under the action of pure internal pressure change and compared the finite element solution with the analytical solution and the test solution of the commercial software ANSYS 10.0, and the results were similar. Rafiee R et al. [58] used ABAQUS software to estimate the rupture pressure of a wound composite pressure vessel from two- and multi-scale analyses. As a variational approach to fracture, the phase field method is a special type of smeared/diffusive gradient damage approach. Among them, the fracture phase field model is a non-local damage model, which has made new progress in the damage simulation of composite materials. Bui T Q et al. [61] have written a review on the application of phase field models to composite materials. Zhang et al. [64] used the interface phase field model to simulate both single material cracks and interface delamination of materials under the framework of fracture phase field theory. Composite laminates have multiple failure modes, and Min L et al. [65] proposed a simple way to embed different failure criteria into the PFM. Zhang et al. [66] developed a double-phase field model for complex failure in fiber-reinforced composites. Non-destructive testing refers to the technical means of detecting, measuring, judging and analyzing the tested object using certain physical methods, chemical methods and acoustic methods without destroying the physical yield being detected. For composite materials, non-destructive testing can accurately find the internal defects of materials in a timely manner and provide an important reference for research. The most common nondestructive testing technologies are ultrasonic testing, eddy current testing and so on. Among them, ultrasonic detection is the most widely used technology. By introducing ultrasonic waves into the material to be measured, it uses the characteristics of ultrasonic waves propagating within the material to detect the defects inside the material, such as pores, cracks, etc. Zhou W et al. [67] reviewed the failure analysis and non-destructive testing of composite hydrogen storage vessels.

Generally speaking, the failure modes of composite materials [68] include fiber failure, matrix failure and interlayer separation. Most of the previous analysis methods for fiber-wound composite gas cylinders used methods such as comparing the stress intensity of the fiber layer and the strength of the material under the load of minimum burst pressure to determine the failure situation of the gas cylinder. For example, T.Y. Kam. et al. [69] studied the failure of the first layer of the composite container. Change.R.R [70] analyzed the failure and failure strength of the first layer of composite gas cylinders by combining theory and test, predicted the failure location of the first layer by acoustic emission technology and verified its reliability through tests. In comparison, it was found that the error between the results of the Tsai–Hill failure criterion and the test results was less than 1%. Cohen David et al. [42] studied the effect of resin on load transfer and the change in failure with fiber volume fraction and predicted the bursting pressure of composite containers based on the analysis results. Madhavi [71] predicted the structural performance of wound composite pressure vessels by analyzing failure modes layer by layer. Since then, scholars have conducted a large number of in-depth studies on the evolution of gas cylinder damage at multiple scales and after damage [72,73,74,75,76]. Rafiee R et al. [72] studied the first-ply-failure (FPF) of composite pressure vessels with/without liners comparing the performance of different failure criteria and then used a progressive damage model based on a continuum damage mechanics approach to deterministically predict the burst pressure of the vessels. Paal O [73] and Eachan C et al. [74] explored the effect of stress intensity factors on the failure of pressure vessels through a comprehensive literature review and numerical simulations. Nagumo Y et al. [75] predicted the failure pressure of a filament-wound (FW) cylindrical vessel by coupling the analytical solution to an existing continuous damage mechanics (CDM) model and compared the predicted failure pressure with the results obtained using Christensen’s failure stress criteria. It was proved that the CDM method is necessary to predict the final failure stress of filament-wound gas cylinders. Lin S et al. [76] used finite element analysis (FEA) to calculate the degradation elastic parameters of composite materials, including fiber and matrix failure modes, and obtained macroscopic stiffness degradation parameters. In addition, the Parker failure criteria were used to simulate the multi-scale progressive damage of composite layers in hydraulic blasting tests.

After the autofrettage pressure treatment, the stress of the liner of a composite cylinder under working pressure was significantly reduced because it was already in the yield state before the treatment. Based on the analysis of the autofrettage pressure treatment and damage factors of the carbon fiber-wound aluminum alloy inner liner composite gas cylinder, the finite element model of the fiber-wound aluminum alloy inner liner composite gas cylinder was established based on the ABAQUS software. Firstly, the influence of autofrettage treatment on the bearing capacity of the gas cylinder was analyzed, and then the damage of the composite material layer under the minimum blasting pressure of the gas cylinder was analyzed. Using the Hashin failure criterion as the failure criterion of the composite material, the failure analysis of fiber-wound composite gas cylinders can observe the initial location of damage. The above research work can provide guidance for the related design, optimization and inspection of gas cylinders.

## 2. Stress Analysis

### 2.1. Modeling of Filament Wound Composite Gas Cylinder

**Figure 1:**
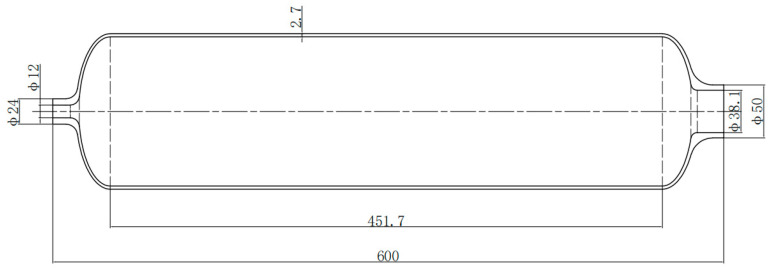
Dimensions of aluminum alloy liner (unit: mm).

A type III composite pressure vessel with a design working pressure of 35 MPa and an aluminum alloy inner container, and its basic dimensions, is shown in Figure 1. The volume of the composite pressure vessel is 9 L. According to DOT-CFFC [77], the design pressure must be greater than 119 MPa. The hydraulic test pressure is generally 1.5 times the nominal working pressure.

The winding of the cylinder in the fiber layer is performed by continuously winding two circumferential winding layers and two spiral winding layers. At the dome, there is no circumferential winding fiber layer. According to the radius of the pole hole and the radius of the barrel and the following formula, the winding angle of the composite vessel is 18°. According to the research, the combination of spiral winding and circumferential winding can make the gas cylinder have stronger carrying capacity and longer fatigue life, so the winding layer of the composite pressure vessel is designed to be [$90_{2}^{\circ }$/$18_{2}^{\circ }$/$90_{2}^{\circ }$/$18_{2}^{\circ }$/$90_{2}^{\circ }$/$18_{2}^{\circ }$/$90_{2}^{\circ }$/$18_{2}^{\circ }$/$90_{2}^{\circ }$/$18_{2}^{\circ }$]. In addition, different winding angles and different layer thicknesses at the dome are modeled in layers.

(1) $$\alpha \left(R\right)=\sin^{-1}\left(\frac{R_{i}}{R}\right)$$

where $R_{i}$ is radius at the dome tangent line; R is the outer radius of the inner bladder cylinder. The fiber thickness of composite pressure vessels is generally calculated using the grid theory. In order to improve the bearing capacity of the head, the fiber winding stress balance coefficient proposed by Chen [6] is introduced. According to the calculation, under the minimum burst pressure, the thickness of the spiral- and circumferentially wound fiber in the cylinder body is the circumferentially wound layer. The thickness of a single layer is 0.217 mm according to the calculation, and the thickness of a single layer of the spiral-wound layer is 0.133 mm.

(2) $$t_{f\alpha }=K\frac{RP_{b}}{2\delta \sigma_{b}\cos^{2}\alpha_{0}}$$

(3) $$t_{f\theta }=\frac{RP_{b}}{2\sigma_{b}}\left(2-\tan^{2}\alpha_{0}\right)$$

where $t_{f\alpha }$ is the total thickness of spiral winding layer; $t_{f\theta }$ is the total thickness of the circumferential winding layer; R is the outer radius of the inner container body; $P_{b}$ is the minimum pressure; *K* is the strength reinforcement coefficient in the range of 1.05–1.4; δ is the stress balance coefficient; and $\sigma_{b}$ is the tensile strength of composite materials.

According to the actual situation, the thickness of the spiral layer on the dome part gradually increases near the pole hole and then rises sharply. The thickness of the spiral layer on the dome section is calculated according to the following formula [78].

(4) $$t\left(r\right)=\frac{t_{tl}\cdot \cos\left(\alpha_{tl}\right)}{\cos\left(\alpha_{r}\right)}\cdot \frac{R_{i}}{r+2\cdot BW\cdot {\left(\frac{R_{i}-R_{0}}{R_{i}-R}\right)}^{4}}$$

where $t_{tl}$, thickness at the tangent line; $\alpha_{tl}$, wind angle at the tangent line; $\alpha_{r}$, wind angle at radius; $R_{i}$, radius at the dome tangent line; $R_{0}$, the radius of the pole hole; R, the outer radius of the inner container body; and BW, helical band width.

**Figure 2:**
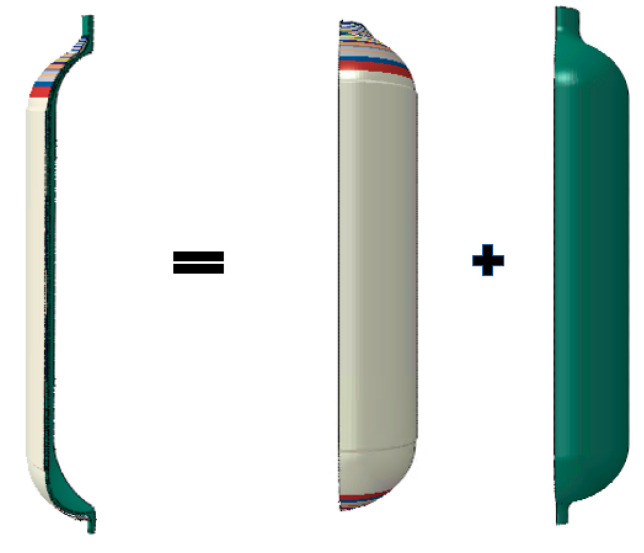
Finite element model of composite pressure vessel.

In this paper, ABAQUS is used to establish the finite element model of the composite pressure vessel. The calculation modeling is as follows: In order to save calculation time, a one quarter symmetric model is established for the finite element calculation. The blue part in Figure 2 is the inner liner, and its thickness is 3 mm.

**Figure 3:**
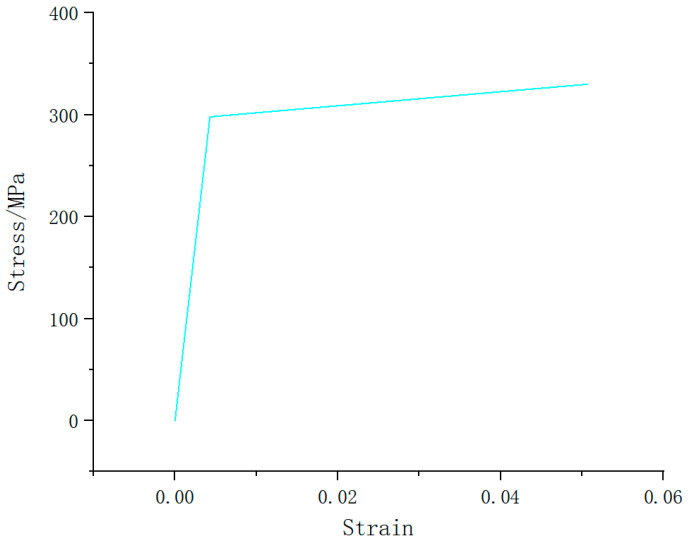
Bilinear model of aluminum alloy material.

The liner is made of aluminum alloy T6061. Its Young’s modulus is 69,000 MPa, Poisson’s ratio is 0.324, the yield stress is 298 MPa and the tangent modulus is 690 MPa. In this paper, the aluminum alloy liner is used as an isotropic elastic–plastic material, and the plastic part is simulated by a bilinear material hardening model, as shown in Figure 3.

The filament winding layer is made of T700/epoxy resin. T700 is carbon fiber and the matrix is epoxy resin. The band width is 3 mm. The mechanical properties of the filament-wound layer are shown in Table 1. The material properties are measured in experiments. E1, E2 and E3 are the Young’s modulus of the fiber layer in the XYZ direction, respectively, and v and G are Poisson’s ratio and shear modulus, respectively. Xt is the fiber tensile strength; Xc is the fiber compression strength; Yt is the matrix tensile strength; Yc is the matrix compression strength; Sxy is the shear strength, Gft is the energy release rate of fiber tensile failure, Gfc is the energy release rate of fiber compression failure, Gmt is the energy release rate of matrix tensile failure, Gmc, is the energy release rate of matrix compression failure and Gs is the energy release rate of shear failure. According to the calculation, each element in each fiber layer is assigned a material type according to the existing user subroutine.

**Figure 4:**
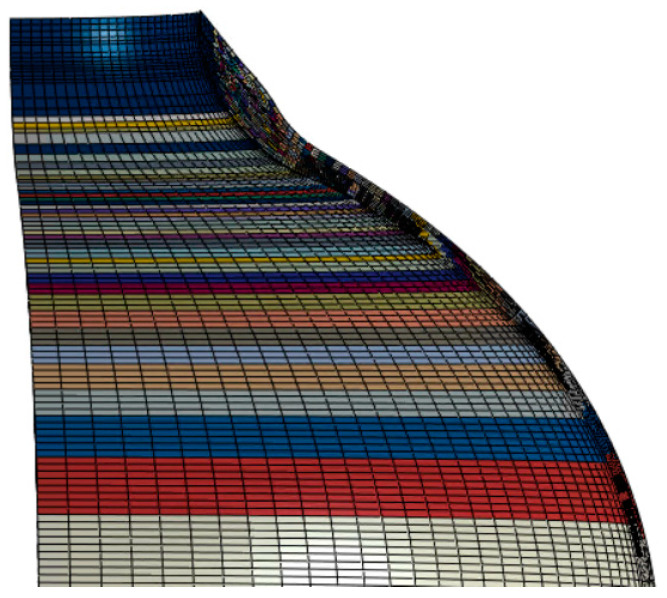
Material properties set according to different winding angles.

The calculation of the orthotropic material properties is performed by first creating the corresponding winding angle for each orthotropic material. That is, all the elements assigned to a given orthotropic material are grouped together according to winding angles. The local winding angle of each element is calculated from the above formula and is determined based on the centroid coordinates projected vertically from the layer to the element at the bottom of the layer. The winding angle range is selected as the spiral winding angle of the cylinder to 90°, and the incremental change in winding angle is selected to divide the grid and assign attributes. For example, this article will create 72 material properties from 18° to 90° degrees in winding angle increments of 1°. The winding angle in the barrel is 18°, and the winding angle of the head at the pole hole is 90°. The angle in the middle is constantly changing. As shown in Figure 4, each color represents a direction, and the change in color at the head represents the change in the winding angle.

**Figure 5:**
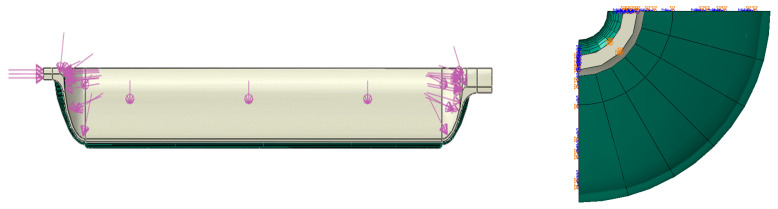
Boundary conditions of filament-wound composite gas cylinder.

The boundary condition of the finite element model of the composite gas cylinder is determined by the structure of the cylinder and the actual working condition. For the one-quarter finite element model in this paper, the displacement boundary condition of symmetrical constraint is imposed on the one-quarter section of the cylinder, the axial displacement constraint is imposed on the cylinder mouth and the uniform load is imposed on the inner surface of the liner. The outer end face of the cylinder mouth is subjected to tensile stress $P\frac{d_{i}^{2}}{d_{0}^{2}-d_{i}^{2}}$ ($d_{i}$, $d_{0}$ are the inner and outer diameters of the gas cylinder mouth, respectively). The boundary conditions of the model are shown in Figure 5.

In the ABAQUS software, the fiber winding layer of the gas cylinder model contains 10 circumferential winding layers and 10 spiral winding layers. The three-dimensional solid unit C3D8R is used in the cylinder part and the C3D8H unit is used in the fiber winding layer. Finally, the inner liner is divided into 30,150 solid units, and the fiber winding layer is divided into 418,800 units.

### 2.2. Autofrettage Stress of Filament-Wound Composite Gas Cylinder

Due to the thin inner liner of the carbon fiber-wound composite cylinder, the bearing capacity of the whole cylinder is not high. The inner liner strength limit is about one-tenth of the outer fiber, and the outer fiber has a high carrying capacity, but it is not fully utilized. In order to improve the overall carrying capacity of the cylinder, the industry often applies pretension pressure inside the cylinder after manufacturing. Under the action of autofrettage pressure, the maximum Mises stress of the inner liner exceeds the yield limit of the inner liner material, and part of the inner liner produces plastic yield. When pressure is applied again, the elastic strain and residual strain of the inner liner are superimposed, which reduces the stress of the inner liner under working conditions. Most of the internal pressure load is borne by the fiber winding layer, thus changing the stress distribution between the fiber layer and the inner liner and improving the life cycle of the gas cylinder. When the autofrettage pressure is large enough, the Bauschinger effect [79] will also occur in the inner layer after pressure relief, resulting in residual compressive stress, while the outer fiber layer will produce certain tensile stress. In this case, when the cylinder is working normally, the tensile stress generated by the inner liner under working pressure and the residual compressive stress generated after autofrettage are superimposed, the maximum stress of the inner liner is reduced and the stress of the outer fiber is increased, which make the stress distribution in the whole wall thickness direction from the inner liner to the outer fiber layer uniform and also improve the bearing capacity of the cylinder.

**Figure 6:**
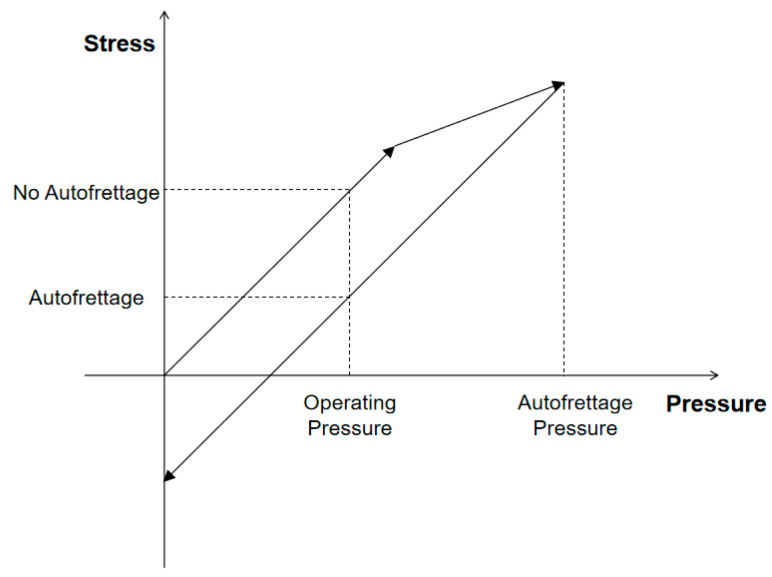
Autofrettage process.

The autofrettage process is shown in Figure 6. Firstly, a force higher than the nominal working pressure is applied to the gas cylinder, which is called autofrettage pressure. In this process, the lining enters the yield deformation stage. Then, the gas cylinder pressure is gradually discharged, and after unloading the metal lining will produce residual plastic strain and the fiber winding layer will produce residual tensile stress. When the pressure of the cylinder is applied to the working pressure again, the elastic strain of the lining is superimposed with the residual strain, which reduces the stress of the metal lining in the working state.

**Figure 7:**
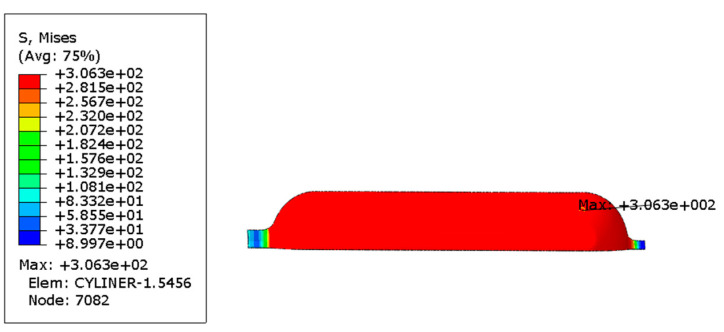
Mises equivalent stress distribution of inner liner under autofrettage pressure (unit: MPa).

**Figure 8:**
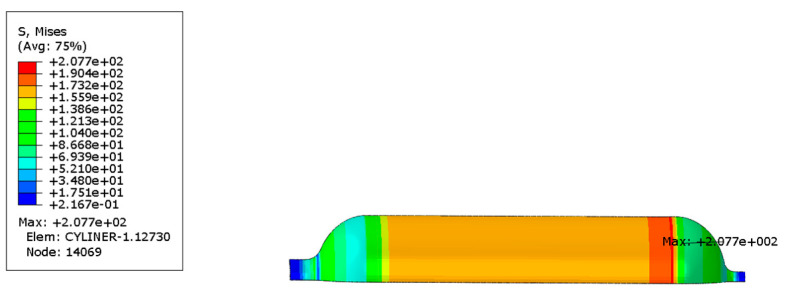
Mises equivalent stress distribution of inner liner at zero pressure after autofrettage pressure (unit: MPa).

In order to investigate the influence of autofrettage pressure on the bearing capacity of gas cylinders, the following two control groups were set for comparison: autofrettage pressure applied before the working pressure and no autofrettage pressure applied before the working pressure. Figure 7 and Figure 8 show the Mises stress distribution under working pressure in the inner liner under the conditions of applying autofrettage pressure and no autofrettage pressure obtained by finite element analysis.

For the convenience of comparison, the Mises stress, the fiber layer circumferential and spiral stress of the inner liner under working pressure, hydrostatic test pressure and minimum burst pressure are listed in Table 2 and Table 3 under no autofrettage pressure and autofrettage pressure.

**Figure 9:**
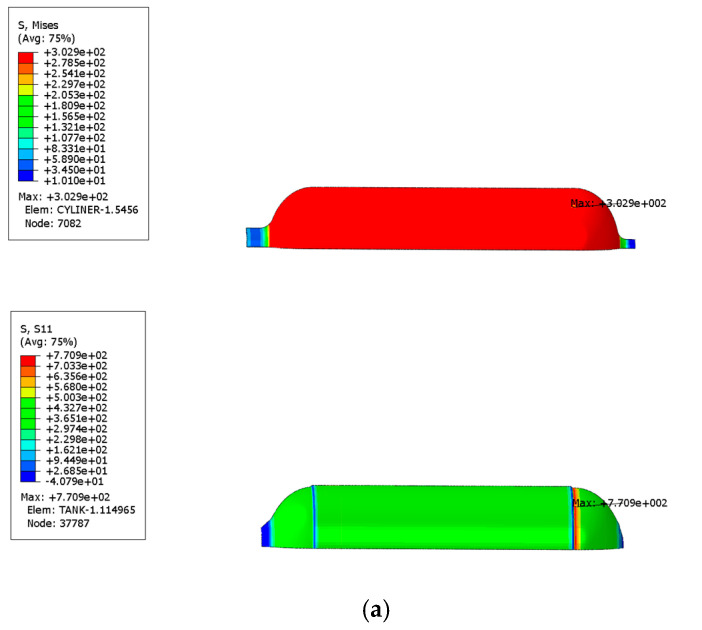

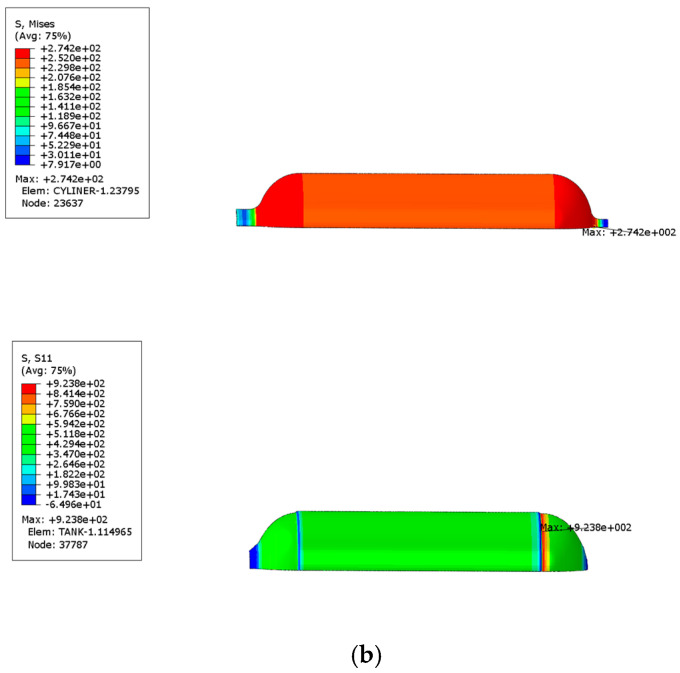
Mises equivalent stress distribution of liner and first principal stress distribution of fiber layer under working pressure (unit: MPa): (**a**) under the action of no autofrettage pressure, (**b**) under the action of autofrettage pressure.

The Mises equivalent stress distribution of the inner liner and the first principal stress distribution of the fiber layer under working pressure when no autofrettage pressure was applied to the gas cylinder are shown in Figure 9a. The Mises equivalent stress distribution of the inner liner and the first principal stress distribution of the fiber layer under working pressure when the autofrettage pressure was applied are shown in Figure 9b. It can be seen from the figure that the maximum Mises equivalent stress of the inner liner decreased from 302.9 MPa to 274.2 MPa under working pressure by applying the autofrettage pressure. As can be seen from Figure 9, the maximum first principal stress of the circumferential fiber layer increases from 1045 MPa to 1328 MPa under working pressure. It can be seen that applying pretension pressure can reduce the stress level of the inner liner under working pressure, while increasing the stress of the fiber layer, that is, applying pretension pressure can improve the stress distribution between the inner liner and the fiber under working pressure.

As can be seen from Table 4, under the condition of applying autofrettage pressure, the inner liner stress under working pressure and hydraulic test pressure is significantly reduced, the fiber stress in the circumferential layer and the spiral layer is significantly increased, the fiber strength is still within the elastic range and the high strength of carbon fiber is fully utilized, and the fiber utilization rate is higher. Therefore, in order to improve the utilization rate of the fiber, the fiber thickness of the circumferential layer can be greater than that of the spiral layer, or the number of fiber layers of the circumferential layer can be greater than that of the spiral layer when designing the carbon fiber layer of the composite cylinder. Since the damage model adopted in this paper did not include the damage evolution part and only used the Hashin criterion to judge the initiation of cracking of the fiber layer, and considering the fact that the fiber layer did not have stiffness reduction processing after failure, there were high-stress data acquired later. Because the minimum blasting pressure exceeds the autofrettage pressure, the inner liner stress and fiber layer stress do not change significantly under the minimum blasting pressure compared with no autofrettage pressure.

**Figure 10:**
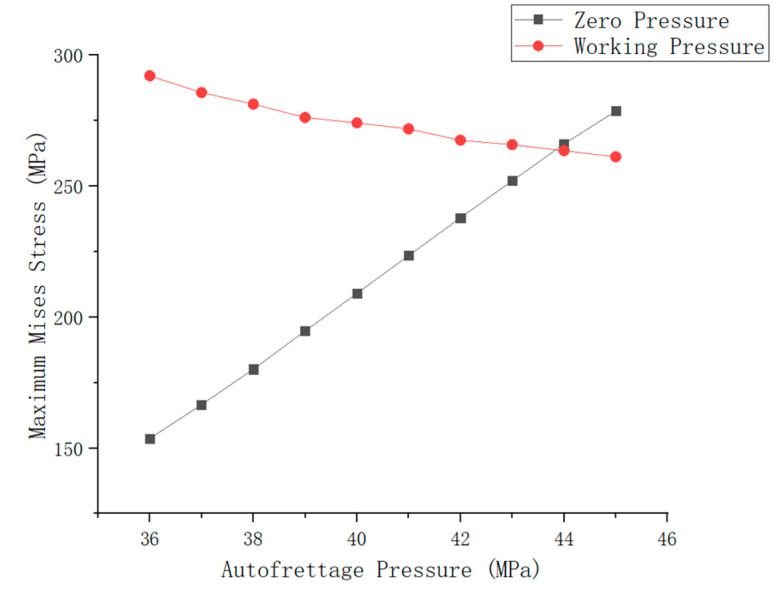
Mises equivalent stress distribution of liner under zero pressure and working pressure.

**Figure 11:**
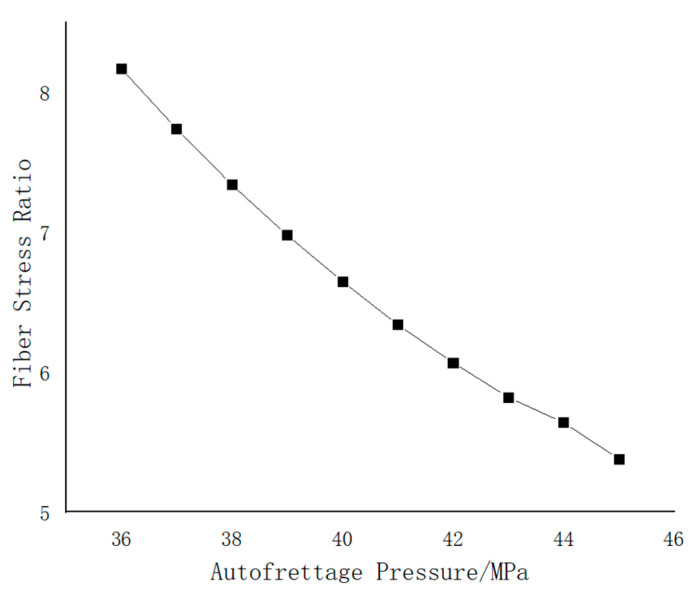
Variation curve of fiber stress ratio with autofrettage pressure.

In order to determine the range of autofrettage pressure, 10 groups of different autofrettage pressures were selected to calculate the maximum Mises stress of the inner liner under zero pressure after autofrettage pressure removal and the maximum Mises stress of the inner liner and fiber stress ratio under working pressure after autofrettage pressure removal, respectively, as shown in Figure 10 and Figure 11.

In Figure 10, the black lines indicate the Mises stress at zero pressure after the removal of different autofrettage pressures. According to the relevant standards, the Mises stress should be greater than 60% (177.6 MPa) of the yield limit of the material and less than 95% (281.2 MPa) of the yield limit of the material, so the autofrettage pressure should be selected in the horizontal coordinate range of 38~44 MPa. The red line shows the Mises stress of the inner liner under working pressure after the removal of different autofrettage pressures. In the relevant standards, the fiber stress ratio is also an important factor in judging the design of gas cylinders. The fiber stress ratio refers to the ratio of the fiber stress under the minimum burst pressure of the gas cylinder to the fiber stress under the nominal working pressure. As can be seen from Figure 11, the fiber stress ratio gradually decreases with the change in autofrettage pressure, so the fiber stress ratio should be greater than 10/3. According to the curve, the autofrettage pressure is 44 MPa.

## 3. Failure Analysis of Cylinder

The strength failure criterion used in the wound composite gas cylinder is used to judge whether the wound composite layer will fail due to damage. The strength failure criterion of the composite material refers to the criterion of judging the material failure caused by external force or the factors determined by the inherent properties of the material itself. In this paper, the carbon fiber composite used in the composite winding gas cylinder is an anisotropic material. Because its strength in the fiber direction is much greater than that perpendicular to the fiber direction, and its tensile strength and compressive strength are also different, it is necessary to distinguish the failure of different directions and tension and compression. The failure criterion used in this paper is the Hashin failure criterion.

The Hashin failure criterion divides the failure of composite materials into two modes: one is based on fiber failure and the other is based on matrix failure. The Hashin invalidation criteria are expressed as follows:

Fiber tensile failure ($\sigma_{1}\geq 0$):

(5) $${\left(\frac{\sigma_{1}}{X_{t}}\right)}^{2}+{\left(\frac{\tau_{12}}{S}\right)}^{2}\geq 1$$

Fiber compression failure ($\sigma_{1}\leq 0$):

(6) $${\left(\frac{\sigma_{1}}{X_{c}}\right)}^{2}\geq 1$$

Matrix tensile failure ($\sigma_{2}\geq 0$):

(7) $${\left(\frac{\sigma_{2}}{Y_{t}}\right)}^{2}+{\left(\frac{\tau_{12}}{S}\right)}^{2}\geq 1$$

Matrix Compression failure ($\sigma_{2}\leq 0$):

(8) $${\left(\frac{\sigma_{2}}{Y_{c}}\right)}^{2}+{\left(\frac{\tau_{12}}{S}\right)}^{2}\geq 1$$

In this paper, the ABAQUS software is used to damage the model of the filament-wound composite gas cylinder under the nominal working pressure of 35 MPa. The final results show that with the increasing stress of the inner pressure winding layer of the cylinder, the fibers and matrix of the cylinder are damaged.

**Figure 12:**
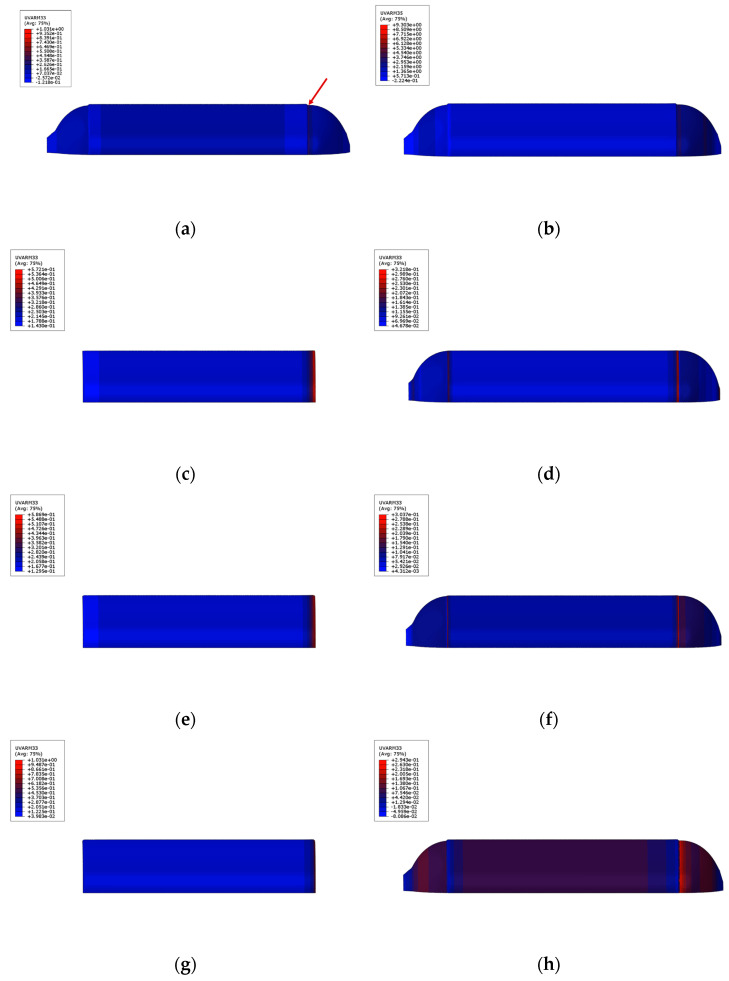
Initial failure contour of the whole fiber winding layer and partial layers. (**a**) Total fiber failure contour; (**b**) total matrix failure contour; (**c**) inner circumferential winding layer; (**d**) inner spiral winding layer; (**e**) intermediate circumferential winding layer; (**f**) intermediate spiral winding layer; (**g**) outer circumferential winding layer; (**h**) outer spiral winding layer.

In this paper, the damage analysis of the fiber-wound composite gas cylinder model is carried out by using the ABAQUS software under a nominal working pressure of 35 MPa. Five analysis steps are set in the whole process: first, loading the internal pressure to the set autofrettage pressure (50 MPa); secondly, setting the unloaded autofrettage pressure (0 MPa); thirdly, loading to the nominal working pressure (35 MPa); fourth, loading to the hydraulic test pressure (52.5 MPa); and lastly, loading to the minimum burst pressure (119 MPa). The gas cylinder damage analysis results calculated by the ABAQUS software are shown in the figure below. According to the results in Figure 12a,b, the matrix damage of the spiral-wound layer first occurs at the joint of the cylinder and the dome. When the internal pressure is 32.123 MPa, the hashin fiber damage criterion reaches 1 and the fibers begin to damage at the point indicated by the arrow. The matrix damage is much larger than 1 at the same time, which proves that the matrix damage had already occurred. The tensile damage first appears in the transition section between the head and the barrel body. By observing the simulation results of the toroidal-wound layer and the spiral-wound layer of the fiber layer, it can be seen from Figure 12c–h that the damage of the toroidal-wound layer first occurs in the outermost toroidal-wound layer and the failure criterion of the inner layer of the spiral-wound layer is larger than that of the outer layer, so it is more prone to damage.

**Figure 13:**
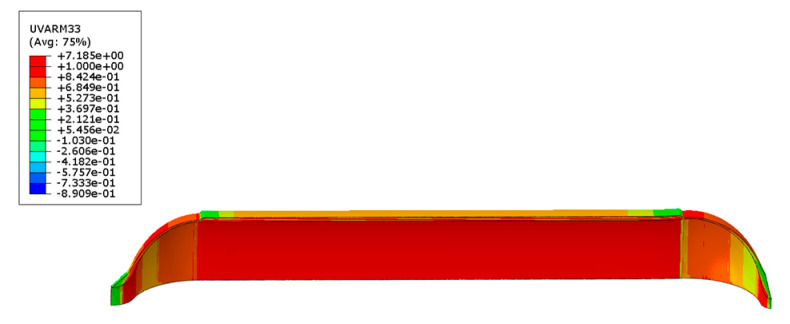
The whole fiber wrapping layer has almost completely failed.

After the hydraulic test pressure, when the pressure reaches 73.6337 MPa, the entire fiber winding layer almost fails, see Figure 13. According to the experimental results, the gas cylinder will burst when the pressure is 42–78 MPa. The simulation results are within the range of burst pressure in the test.

**Figure 14:**
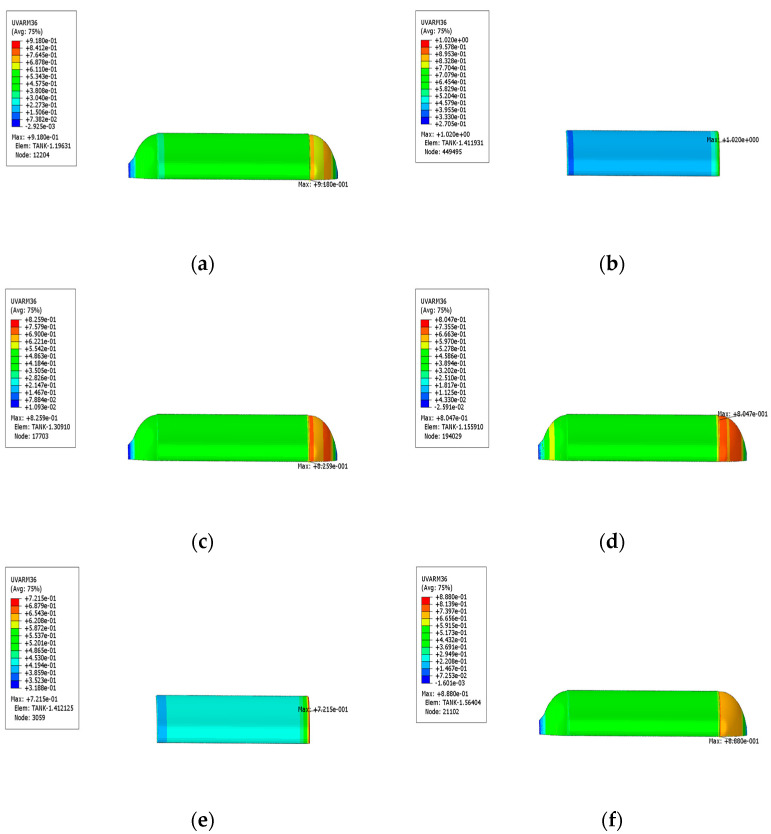

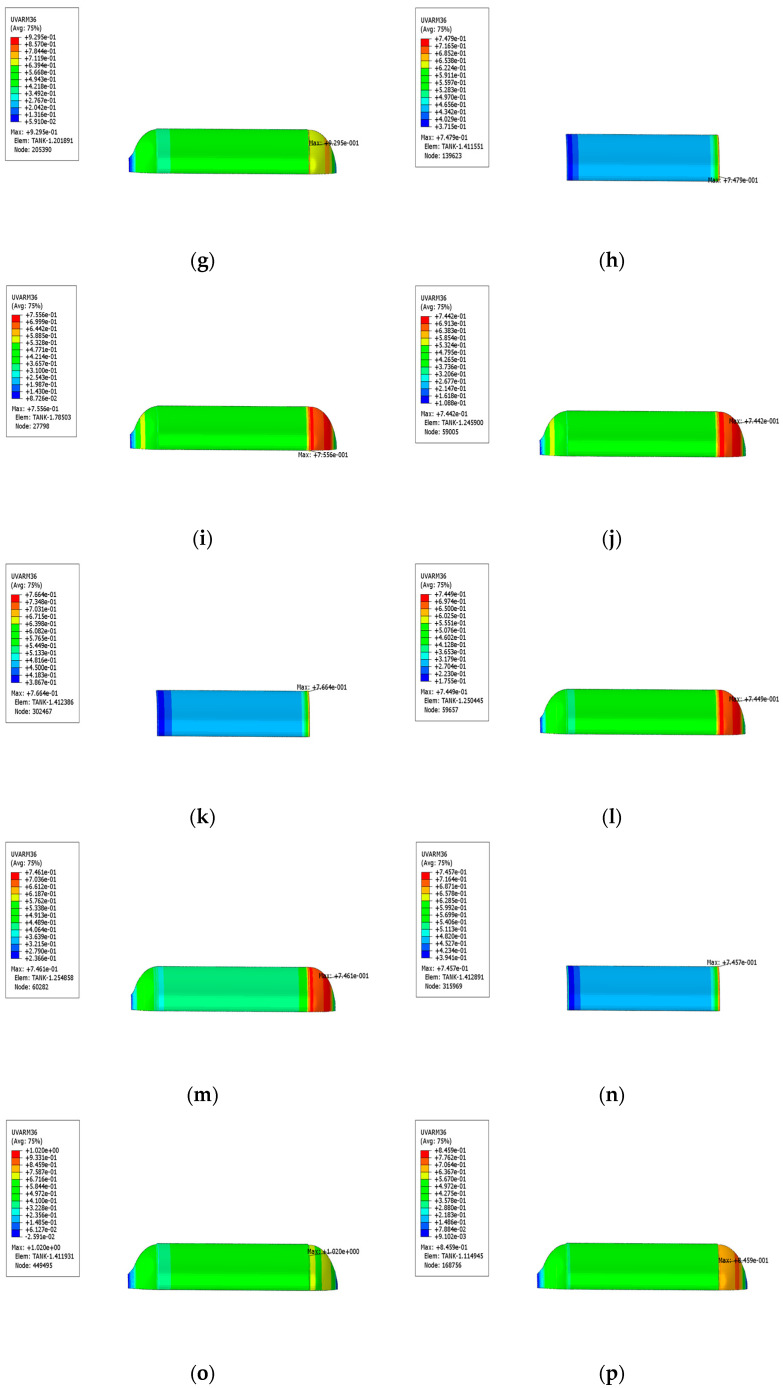
Initial failure contour of the whole fiber winding layer and every layer. (**a**) All layers; (**b**) the first fiber layer; (**c**) the second fiber layer; (**d**) the third fiber layer; (**e**)the fourth fiber layer; (**f**) the fifth fiber layer; (**g**) the sixth fiber layer; (**h**) the seventh fiber layer; (**i**) the eighth fiber layer; (**j**) the ninth fiber layer; (**k**) the tenth fiber layer; (**l**) the eleventh fiber layer; (**m**) the twelfth fiber layer; (**n**) the thirteenth fiber layer; (**o**) the fourteenth fiber layer; (**p**) the fifteenth fiber layer.

The results of Figure 14a show that when the internal pressure is 45.8115 MPa, the damage criterion of the maximum stress criterion reaches 1, and the fiber layer begins to be damaged. As can be seen from Figure 14b–p, the fiber damage in the fiber cladding layer also first appears in the outermost layer, and the circumferential cladding layer is more prone to damage.

## 4. Conclusions

- Starting from the perspective of autofrettage treatment and damage analysis of a fiber-wound composite gas cylinder, the angle and thickness of the fiber layer are calculated by using the grid theory for modeling. The fiber winding method is geodesic winding. The ABAQUS subroutine compiled by the Hashin criteria is used to analyze the damage during the process from working pressure to blasting pressure of the fiber-wound composite gas cylinder.
- The simulation results show that applying autofrettage pressure can reduce the stress level of the inner liner under working pressure and increase the stress of the fiber layer. In other words, applying autofrettage pressure can improve the stress distribution between the inner liner and the fiber under working pressure. In the study, failure analysis was carried out on a fully wound carbon fiber gas cylinder with an aluminum alloy inner liner under nominal working pressure of 35 MPa. According to the simulation results, it was found that the initial damage of the matrix of the fiber layer appeared in the transition section between the dome and the barrel body, and the damage generally started from the circumferential winding layer and the outer layer of the winding layer.

## Figures and Tables

**Table 1:** Mechanical property parameters of T700/epoxy.

Parameter	Value	Parameter	Value
E1/GPa	130	Xt/MPa	2080
E2/GPa	7.7	Xc/MPa	1250
E3/GPa	7.7	Yt/MPa	60
v12	0.3	Yc/MPa	140
v13	0.3	Sxy/MPa	110
v23	0.35	Gft/N/mm	133
G12/GPa	4.8	Gfc/N/mm	10
G13/GPa	4.8	Gmt/N/mm	0.5
G23/GPa	3.8	Gmc/N/mm	1.6
		Gs/N/mm	1.6

**Table 2:** Cylinder loading pressure (included autofrettage).

Autofrettage Pressure /MPa	Zero Pressure /MPa	Working Pressure /MPa	Hydraulic Test Pressure /MPa	Minimum Burst Pressure /MPa
40	0	35	52.5	120

**Table 3:** Cylinder loading pressure (no autofrettage).

Working Pressure /MPa	Hydraulic Test Pressure /MPa	Minimum Burst Pressure /MPa
35	52.5	120

**Table 4:** Comparison of stress under different working conditions with or without autofrettage pressure.

	Working Pressure			Hydraulic Test Pressure			Minimum Burst Pressure		
	Maxi-Mum Mises Stress of Liner/MPa	Maxi-Mum S1 Stress of Circum-Ferential Fiber Layer/MPa	Maxi-Mum S1 Stress of Spiral Fiber Layer/MPa	Maximum MISES Stress of Liner/MPa	Maxi-Mum S1 Stress of Circu-Mfere-Ntial Fiber Layer/MPa	Maxi-Mum S1 Stress of Spiral Fiber Layer/MPa	Maxi-Mum Mises Stress of Liner/MPa	Maxi-Mum S1 Stress of Circumferential Fiber Layer/MPa	Maxi-Mum S1 Stress of Spiral Fiber Layer/MPa
No Autofrett-age Pressure	302.9	1045	772	318.7	2412	1897	330	7201	8380
Autofrett-age Pressure	274.2	1328	925.2	318.7	2413	1564	330	6842	7842

## Data Availability

Data are contained within the article.
